# Development of NIRS method for quality control of drug combination artesunate–azithromycin for the treatment of severe malaria

**DOI:** 10.1016/j.jpba.2012.04.009

**Published:** 2012-08

**Authors:** Chantal Boyer, Karen Gaudin, Tina Kauss, Alexandra Gaubert, Abdelhakim Boudis, Justine Verschelden, Mickaël Franc, Julie Roussille, Jacques Boucher, Piero Olliaro, Nicholas J. White, Pascal Millet, Jean-Pierre Dubost

**Affiliations:** a Développements Analytiques et Pharmaceutiques appliqués aux Maladies Négligées et aux Contrefaçons, EA 4575, Université Bordeaux Segalen, 146 rue Léo Saignat, 33076 Bordeaux Cedex, France; b Tropical Disease Research, World Health Organization, Geneva, Switzerland; c Tropical Medicine, Mahidol University, 420/6 Rayvithi Road, Bangkok 10400, Thailand; d University of Oxford, UK

**Keywords:** Artesunate, Azithromycin, NIRS, Green chemistry, Chemometrics

## Abstract

Near infrared spectroscopy (NIRS) methods were developed for the determination of analytical content of an antimalarial-antibiotic (artesunate and azithromycin) co-formulation in hard gelatin capsule (HGC). The NIRS consists of pre-processing treatment of spectra (raw spectra and first-derivation of two spectral zones), a unique principal component analysis model to ensure the specificity and then two partial least-squares regression models for the determination content of each active pharmaceutical ingredient. The NIRS methods were developed and validated with no reference method, since the manufacturing process of HGC is basically mixed excipients with active pharmaceutical ingredients. The accuracy profiles showed β-expectation tolerance limits within the acceptance limits (±5%). The analytical control approach performed by reversed phase (HPLC) required two different methods involving two different preparation and chromatographic methods. NIRS offers advantages in terms of lower costs of equipment and procedures, time saving, environmentally friendly.

## 1 Introduction

Malaria manifests itself most commonly with fever and other signs and symptoms that are aspecific and can be caused by other infections [1]. Differential diagnosis may be very difficult, even when the IMCI (Integrated Management of Childhood Illnesses) guidelines and algorithms are applied [2]. The diagnosis of malaria requires that parasites are observed microscopically or parasite antigens are detected by means of rapid diagnostic tests. However, such tests may not be available in rural communities (where the majority of cases occurs), and in malaria-endemic areas sometimes even the detection of malaria parasites cannot rule out other infections. Microbiological and clinical diagnosis of bacterial infections is challenging in rural tropics.

Both malaria and bacterial pneumonia and sepsis can progress rapidly if not promptly diagnosed and treated. Children become unable to take medications orally (because of intense vomiting), but injections are generally not available in rural communities or even sometimes in peripheral health facilities (nor are they advisable if safe injections cannot be guaranteed).

Rectal artesunate (AS) prevents malaria progression and reduces malaria mortality [3] when given to children who cannot take oral medications (a condition referred to “moderately severe”, or “non-per-os” malaria) who are further referred to the nearest health facility where appropriate follow-on treatment can be instituted. Similarly, combining AS with an antibiotic would cover both malaria and bacterial infections; if this antibiotic had, like azithromycin (AZ) does, also antimalarial properties [4–7], additional antimalarial effects and protection against resistance would be conferred.

Therefore, we have studied the possibility of developing a rectal, fixed-dose combination of AS plus AZ, as an emergency pre-referral treatment of children living in malaria endemic areas who develop fever and deteriorating conditions and cannot thus be treated orally.

The selected rectal form is Hard Gelatin Capsules (HGC) containing both Active Pharmaceutical Ingredients (APIs) as powder mixture with excipients. As part of this development, we were faced with the challenge of developing a single method for API content determination for quality control.

A single chromatographic method using RP-HPLC with grafted silica is not feasible because of the eluotropic strength and the pH of the mobile phase. We therefore considered a method by high temperature liquid chromatography using non-aqueous mobile phase and porous graphitic carbon as stationary phase hyphenated with evaporative light scattering detection (ELSD) [8] for the elution in the same run of the both AS and AZ. However this method is not sufficiently robust for analytical control purpose due to the poor thermostability of AS associated with less reproducibility of ELSD than UV spectroscopy. However, the quality control of AS and AZ in HGC by RP-HPLC with UV detection required two different methods, and thus two chromatographic equipments to perform the determination content of the two APIs. As HPLC is time-consuming, expensive, labor-intensive and requires solvents, there is a need for a fast, accurate and economical methods for AS and AZ quantification in the HGC.

Near infrared spectroscopy (NIRS) is attractive because it is rapid (direct recording of spectra with little or no sample pre-treatment), produces no chemical waste, and provides chemical and physical information about samples. NIRS is used as a quality control technique in the pharmaceutical industry and represents a useful tool for direct analysis of pharmaceutical samples [9–11].

At the same time, the combination of a chemometric software and the use of multicomponent analytical methods are required to interpret the large amount of information present in NIR spectra. Partial Least Squares (PLS) is a regression method frequently used for quantitative purposes in which the calibration involves correlating the data in the spectral matrix X with the data in the concentration matrix Y. PLS needs (i) a calibration set whereby the relationship between the spectra and the concentration of the API is derived, followed by (ii) a validation set for the determination of the API concentration from the spectra of the analyzed samples set according to the calibration results.

The presence of AS and AZ in high concentrations in the formulated product (51.06 and 36.56%, w/w, respectively) compensates for the limited sensitivity of the NIRS technique. Feasibility studies of using NIRS to identify counterfeit drugs containing AS have been reported [12,13] but AS lacks functions suitable for quantitative evaluation. Gabriëls [14] reported a CZE-technique for the separation of artesunate and artelinic acid using micellar electrokinetic capillary electrophoresis with UV-detection at 185 nm. Green [15] evaluated a technique combining refractometry and colorimetry as simple and accurate field assays for rapid assessment of the content of AS tablets. As for AZ, a NIRS identification system has been developed for the analysis of AZ tablets from different manufacturers [16].

The objective of this work was to investigate the suitability of the NIRS technique associated with chemometrics for quantification of AS and AZ in HGC in order to avoid the use of two different HPLC methods. One model for each API was developed, and the analytical methods were validated. The development of the methods was attempted without a reference [17,18] and compared to validation results obtained by HPLC.

## 2 Material and methods

### 2.1 Chemicals and reagents

AS was a generous gift of DND*i* (Drug for Neglected Disease initiative, Geneva, Switzerland) purchased from Knoll BASF Pharma (Liestal, Switzerland). β-Dihydroartemisinin (DHA, AS main metabolite) was synthesized from COMIPSO (Bordeaux, France). AZ was purchased from Pfizer (USA). Microcrystalline cellulose (Emcocell 90, Mendell, Finland) and colloidal silica (Aerosil 300, COOPER, France) were of pharmaceutical grade.

### 2.2 FT-NIRS equipment and recording of NIR spectra

Spectra were acquired on a Thermo Scientific FT-NIR analyser model Antaris II (ThermoScientific Instrument, Madison, USA) controlled by Result and Omnic softwares. This instrument is equipped with a Michelson interferometer, a matched InGaAs detector, a quartz halogen light source (50 W) and an Antaris II Integrating sphere for measuring solids by diffuse reflectance.

The spectrophotometer was equipped with the software package from ThermoScientific Instrument, including OMNIC version 6.1a for spectral acquisition and TQ Analyst version 6.21 for spectral processing and chemometric analysis. All measurements of powder mixtures were collected in the diffuse reflectance mode and spectra were converted in absorbance units. Each powder blend was poured in a 1.5 mL glass vial (Chromacol – 10 mm in diameter – from ThermoScientific Instrument Inc.), put on the sampling sapphire window of the reflectance module and scanned directly through the bottom of the vial (a special holder made to fit the shape of the vial kept the container in centered position over the window). Each spectrum was the average of 32 co-added scans, obtained with usual resolution of 8 cm^−1^ over the range of 4000–9999 cm^−1^ and corrected against the background spectrum of room environment which was performed routinely. Each sample was recorded in triplicate with turnover between successive recordings to obtain an average spectrum.

### 2.3 HGC manufacturing

All components of HGC (Table 1) were mixed in a planetary mixer for 15 min. The powder blend was filled into colorless 000 HGC with semi-automatic capsule filler equipped with vibrator (LGA QB 30, Laboratoire des Gélules et des Azymes, France). The formulation was optimized for its flowing properties. A batch of 1200 HCGs was prepared and tested according to Pharmacopoeia standards.

### 2.4 Development and validation of NIRS model

A Partial Least Squares (PLS) model as provided in the TQ Analyst software was used for the quantification of AS and AZ with two different calibration models.

All data were mean centered before analysis. Spectral pre-treatments tested to construct the calibration models include Standard Normal Variate (SNV) and first Stavisky–Golay derivative with 11-points moving window and fitting to a second-order polynomial. PLS calibration models were constructed and the goodness of fit of the models was assessed in terms of their standard relative errors, i.e. the Root Mean Square Error of Calibration (RMSEC), and Root Mean Square Error of Prediction (RMSEP). The optimum number of PLS factors used to construct the models was the one resulting in the lowest error of prediction for an external set of validation samples, i.e. the Root Mean Square Error Cross Validation (RMSECV). The predictive ability of the model was tested by applying the validation set that was not used during its development.

For the NIRS calibration method development, for each API, four sets of reconstituted powder blends were performed at API concentrations ranging from 80 to 120% of the labeled concentration of the API of interest in HGC (the concentration of the other API is fixed at 100%). Sets of calibration and validation for the models development were performed as described in Tables 2 and 3, for AZ and AS, respectively. Before weighing, the required component was individually sieved to obtain a granulation under 250 μm like in the HGC formulation process. 2053.6 mg and 1026.82 mg of each blend were prepared for the calibration and validation sets, respectively. The resulting mixtures were poured in test tubes without grinding and serially mixed with manual turbula during 1 minute in order to ensure the homogeneity of the material. Then each powder blend was aliquoted in 1.5 mL glass vials to reach 5 mm height of powder which corresponded to around 200 mg of the mixtures. 6 and 3 vials for each concentration level were used for calibration and validation model development, respectively.

### 2.5 Validation of NIRS methods

For the specificity study of the NIRS methods, excipients mixture (placebo) was prepared by weighting and mixing together 4998.9 mg of microcrystalline cellulose and 122.7 mg of colloidal silica.

A mixture labeled “10% DHA” consists of 261.88 mg of AZ, 168.75 mg of AS, 18.75 mg of DHA, 64.04 mg of excipients mixture (i.e. placebo).

For the validation of the NIRS analytical methods, a new set of samples for each API was prepared as previously described, with one API content varying while keeping the other one fixed (and vice versa). Each set was composed of three levels (80, 100 and 120%) of the API labeled concentration, and three independent replicates of this set of samples were prepared and analyzed on three different days by two different persons to establish accuracy profiles.

### 2.6 HPLC equipment and procedures

The HPLC system was a Hewlett Packard HPLC 1050 series consisting of a quaternary pump, a DAD detector, an online degasser, an Agilent Chemstation LC 3D, a Rheodyne 7125 injection valve (Cotati, California, USA) with injection volume at 5 μL.

The HPLC method for AS determination [19] consisted of a column X-Terra RP C18 (50 mm × 3 mm i.d., 3.5 μm particle size) Waters (Milford, MA, USA) used with CH_3_CN–HCOOH (pH 2.85; 0.01 M) (40:60, v/v) as mobile phase, thermostated at 60 °C and flow rate at 0.7 mL min^−1^. The detection was set at 220 nm. The HPLC method for AZ determination [20] consists of a column Luna C8 EC 5 μm, 150 mm × 4.6 mm (Phenomenex, France) used with CH_3_OH–phosphate buffer (pH 7.5; 30 mM) (80:20, v/v) (pHws=10) as mobile phase, thermostated at 20 °C and flow rate at 1 mL min^−1^. The detection was set at 210 nm.

For sample preparation for HPLC, twenty capsules were milled. Then 274 mg and 196 mg of this blend for AS and AZ content measurements, respectively, were weighted, dissolved in 20 mL-volumetric flask with methanol, filtrated with 0.45 μm-filter and finally diluted 10-fold in respective mobile phase.

### 2.7 HPLC validation methods

For the validation of the HPLC method procedure, two sets of three levels (80, 100 and 120%) of the API labeled concentration were prepared, one composed only with API and the other composed by samples of reconstituted forms.

For the reconstituted form of 100% level of AS HPLC procedures, 100 mg of AS with 139.7 mg of AZ and 34.1 mg of the placebo were weighted, dissolved in 20 mL-volumetric flask with methanol, filtrated with 0.45 μm-filter and finally diluted 10-fold in mobile phase. Then only the weight of AS varied for 80 and 120% levels at 80 and 120 mg, respectively. For each of the three levels of reconstituted form and API sets, 5 and 3 different solutions were prepared, respectively.

For the reconstituted form of 100% level of AZ HPLC procedures, 100 mg of AZ with 71.6 mg of AS and 24.4 mg of the placebo were weighted, dissolved in 20 mL-volumetric flask with methanol, filtrated with 0.45 μm-filter and finally diluted 10 fold in mobile phase. Then only the weight of AZ varied for 80 and 120% levels at 80 and 120 mg, respectively. For each of the three levels of reconstituted form and API sets, 5 and 3 different solutions were prepared, respectively.

## 3 Results and discussion

### 3.1 Calibration models development

For method development in NIRS without a reference method, the parameters of the pharmaceutical process must be taken into account. Granulation and milling for the HGC formulation may affect NIR spectra. Reliable dosage requires a uniform distribution of the API and the excipients in the pharmaceutical blend; only the first 0.75 mm layer of a powder blend is analyzed with diffuse reflectance spectroscopy and the analysis depends on particle size [21]. In order to obtain the most representative spectrum from each sample, the individual component powders were granulated with a similar particle size (∼250 μm) as in the formulation process. After mixing, identical amounts of the blend were poured in vials before acquisition of the spectra, which led to equivalent powder height for NIRS light path (∼5 mm). For each concentration, three spectra obtained after successive turnarounds were averaged to give a mean spectrum for each sample. The time of manual turbula mixing was increased to 1 min, as the time at which the mixture became uniform with consistent spectra obtained from different vials.

**Fig. 1:**
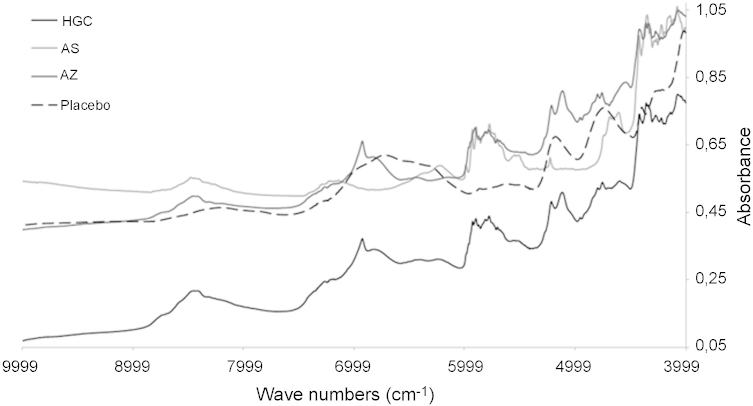
Spectra of pure compounds AS and AZ, Placebo (excipients mixture) and HGC.

The spectrum of an HGC sample is dominated by the AZ spectral features (Fig. 1). AZ represents 51.06% (w/w) in the capsule and its spectrum shows distinct spectral differences from that of placebo. So the PLS regression which can correlate NIRS data with API concentrations was applied in spectral ranges which involved the largest differences between placebo and HGC spectra, i.e. two regions ranging from 5311 to 6811 and from 7200 to 9999 cm^−1^.

The development of a NIRS model consists in checking different spectral pre-treatments as well as its combination with different spectral ranges. Among the most widely used pre-processing techniques, SNV [22] reduces physical variability, adjusts for baseline shifts between samples, minimizes the spectral contribution of differences in particle size and does not increase noise in the signal [23]. As for first or second spectral derivative, these treatments can remove both additive and multiplicative effects in the spectra, thus reducing spectral contributions from the physical properties of samples. All these pre-processing approaches were tested with a view to constructing the calibration models. At the end of this process, the combination of SNV pre-treatment associated with the first derivative transformed spectra and the raw spectra over the respective spectral range 5311–6811 and 7200–9999 cm^−1^ was the one providing the best results in all instances.

In order to allow for independence between respective API concentrations, two different models were created for AS and AZ quantification, respectively. For each model, calibration and validation sets consisted of 6 and 3 individual mixtures, respectively, at each level. As the sets contained nine levels, the calibration and validation sets consisted respectively of 54 and 27 samples.

The external validation of the method was performed with the samples of the validation set, i.e. samples that are independent from those used to calibrate the method. It was done in parallel with the threshold setting, to ensure a correct selection of this parameter. Once the calibration models were defined, their predictive ability was tested using the validation set. The calibration models were chosen in terms of predictive ability and prediction errors (Table 4). The selected strategies allowed models to be constructed from a number of PLS significant factors, i.e. 5 and 6 for AZ and AS, respectively. The determination coefficient values were close to 1 for each model. RMSEP and RMSEC values were weak, with RMSEC smaller than RMSEP. These models displayed excellent results in terms of merit values at the calibration and validation stages. Each interval error never exceeded the acceptance threshold. So the PLS method was validated before its application for quantitative determination of AZ and AS in different HGC samples.

### 3.2 Validation

The validation of the NIRS measurements associated with multivariate analyses was considered to fulfill the expectations of ICH Q2 (R1) guideline on validation of analytical procedures [24].

#### 3.2.1 Specificity

**Fig. 2:**
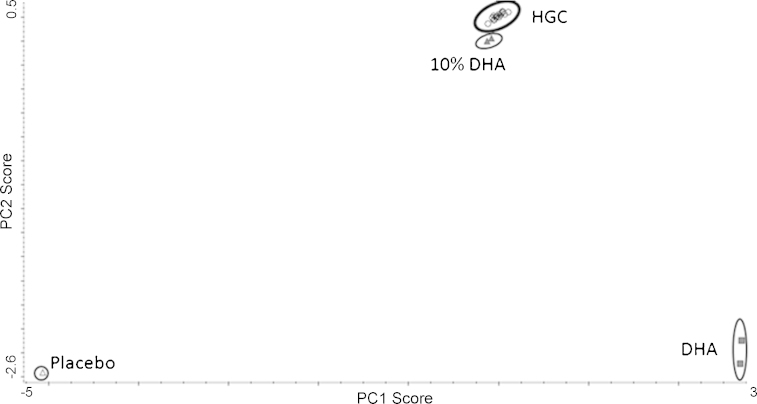
Scores plot resulting from a PCA of the API specific region (5311–6811 and 7200–9999 cm^−1^). Spectra were treated with combination of SNV pre-treatment associated with the first derivative transformed spectra and the raw spectra, respectively of each spectral range. Percentage of variance explained by PC1 and PC2 = 99.994%.

For NIRS methods, applying the PLS model is not enough per se to ensure method specificity. To test the specificity of the methods for APIs quantification, potential challenges are presented to the library. The specificity proof-set included placebos, degradation product (i.e. DHA) of AS, HGC formulations from the laboratory pharmaceutical production. Degradation of AS occurred by interaction with excipients, other APIs or humidity [25]. The library rejected all the samples from the specificity proof-set, which was taken as evidence of its specificity. Fig. 2 presents a scores plot from a PCA where the specificity proof-set was included. In this plot, the distance of the different products from the HGC samples shows that this identification method clearly discriminates different products.

#### 3.2.2 Accuracy profiles

**Fig. 3:**
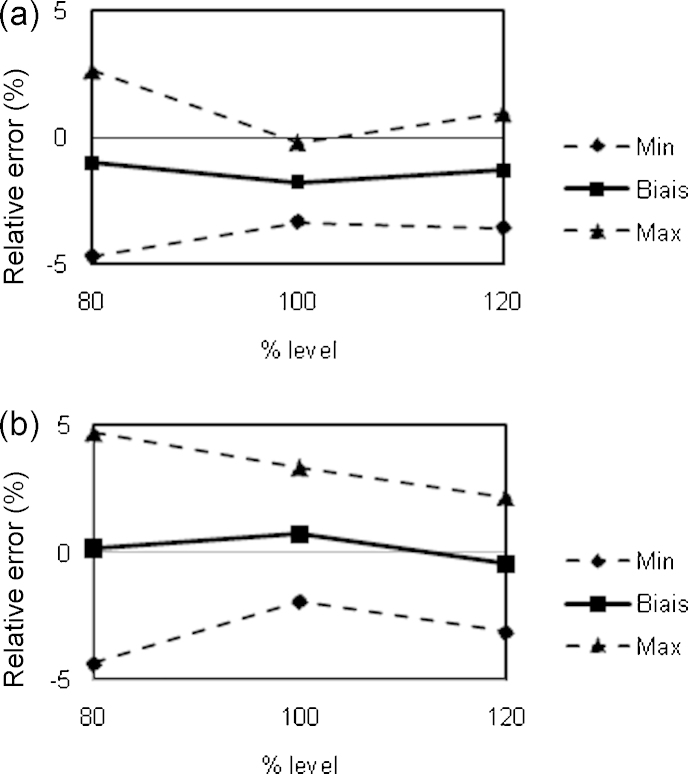
Accuracy profiles from the validation of HPLC (a) and NIRS (b) methods for AS determination content. Acceptance limits at ±5%.

**Fig. 4:**
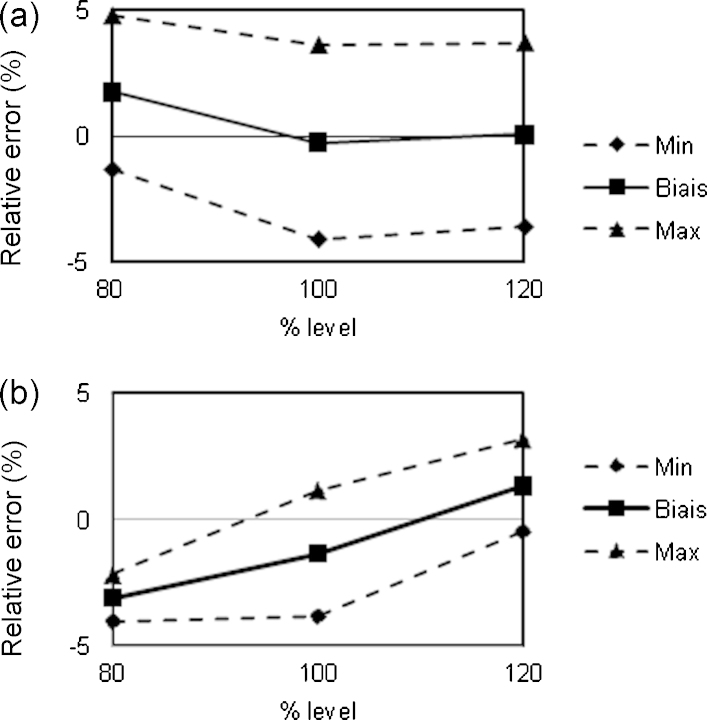
Accuracy profiles from the validation of HPLC (a) and NIRS (b) methods for AZ determination content. Acceptance limits at ±5%.

The validation of the analytical methods was done using the accuracy profiles approach [26–31]. The accuracy of an analytical procedure expresses the agreement between the experimental and the conventional true value or an accepted reference value. Accuracy represents the sum of systematic (trueness) and random errors (precision) and therefore, expresses the inherent total error of the result. For a pharmaceutical formulation, acceptable limits were fixed at ±5%. As shown in Figs. 3 and 4, β-expectation tolerance limits obtained with NIRS approaches were within in these limits.

### 3.3 Comparison between NIRS and HPLC methods

HPLC required two independent methods for determination of the content of each API. The whole analytical procedures in terms of sample preparation and chromatographic conditions were different. The NIRS approach required the record of a single spectrum which required only the opening of the HGC. Then APIs content determination by NIRS was performed using one mathematical model for each API.

The NIRS and HPLC methods were validated within the ±5% acceptance limits (Figs. 3 and 4). The validation performance obtained for each API was equivalent between NIRS and HPLC. However, with HPLC, the accuracy profiles of both APIs were obtained within the ±5% acceptance limits with regression model between the response *R* and the concentration *C* following linear relationship *R* = *aC* + *b* where a and b are constant values. Therefore the use of the HPLC methods for analytical control purpose required additional concentration calibration before daily content determination. In case of NIRS methods, as one PLS model was validated for each API, no further analysis was required for the content determination of APIs in the HGC.

Each HPLC method had a run time of 3 and 9 min for AS and AZ, respectively. It should also be noted that analyzing AS by HPLC required extemporaneous sample preparation to ensure AS stability. Further, RP-HPLC approaches were time-consuming, required extensive sample preparation and consumed large quantities of solvent especially in the case of AZ where the mobile phase required 80% of organic solvent.

The AS and AZ content measurements by the two different instruments were performed on a fresh batch. The results obtained from 6 determinations were 103.3% (RSD 2.6%) and 104.9% (RSD 3.5%) for AS, and 102.1% (RSD 1.7%) and 103.9% (RSD 4.4%) for AZ, with NIRS and HPLC methods, respectively. Similar results were thus obtained with an additional advantage of the NIRS instrument which is less expensive to purchase and operate than a standard HPLC system.

## 4 Conclusion

The proposed NIRS methods were validated for the determination of content of two APIs (AS and AZ) in the same pharmaceutical form. As the form is HGC, the pharmaceutical process was very simple and allows developing a chemometric model with no reference method, making also both the entire analytical development and sample preparation procedure environmentally friendly. In addition, using this approach instead of HPLC, circumvented the problem of API stability during sample preparation especially for AS, saved time, reduced considerably workload, minimized the consumption of disposable material and was thus economically competitive.

## Figures and Tables

**Table 1:** Azithromycin/Artesunate formulation composition in HGC.

Composition	HGC content	
	mg per HGC	%w/w
Azithromycin, 2H_2_O	419	51.06
Artesunate	300	36.56
Microcrystalline cellulose	100	12.18
Colloidal silica	1.638	0.20

**Table 2:** Weight of the different HGC components for the AZ calibration and validation models (AS fixed).

%	Calibration set				Validation set			
	AS (mg)	AZ (mg)	Placebo^a^ (mg)	Total (mg)	AS (mg)	AZ (mg)	Placebo^b^ (mg)	Total (mg)
80	836.6	750.0	465.2	2051.8	418.6	375.5	232.4	1026.5
85	889.1	749.6	413.5	2052.2	445.5	375.2	205.8	1026.4
90	942.5	750.6	360.6	2053.7	471.5	375.4	180.2	1027.1
95	993.5	750.5	308.2	2052.2	496.2	375.3	154.5	1026.0
100	1046.5	750.3	256.3	2053.1	523.4	374.9	128.1	1026.4
105	1099.5	750.1	203.4	2053.0	549.2	375.6	101.6	1026.4
110	1150.8	750.2	151.4	2052.4	577.8	375.4	76.1	1029.3
115	1206.4	750.1	98.8	2055.3	602.9	375.6	49.6	1028.1
120	1256.6	750.2	46.5	2053.3	628.3	375.3	23.6	1027.2

a qsp 2053.64 mg.

b qsp 1026.82 mg.

**Table 3:** Weight of the different HGC components for the AS calibration and validation models (AZ fixed).

%	Calibration set				Validation set			
	AS (mg)	AZ (mg)	Placebo^a^ (mg)	Total (mg)	AS (mg)	AZ (mg)	Placebo^b^ (mg)	Total (mg)
80	602.1	1046.5	405.0	2053.6	301.8	535.5	202.9	1040.2
85	637.5	1046.1	368.1	2051.7	319.6	524.1	184.4	1028.1
90	675.6	1047	330.7	2053.3	338.0	523.4	165.5	1026.9
95	712.6	1046.1	293.7	2052.4	356.8	523.0	146.2	1026.0
100	749.9	1045.0	256.4	2051.3	375.6	523.8	127.7	1027.1
105	787.8	1047.3	217.6	2052.7	395.4	522.9	109.2	1027.5
110	824.6	1045.9	181.0	2051.5	412.6	523.1	90.8	1026.5
115	862.3	1047.7	143.5	2053.5	431.9	523.3	71.1	1026.3
120	900.1	1045.5	105.9	2051.5	450.3	523.2	53.5	1027.0

a qsp 2053.64 mg.

b qsp 1026.82 mg.

**Table 4:** Results of PLS models for AZ and AS.

	AZ	AS
Number of factor	5	6
*r*	0.999	0.999
Error	[−2.3; 2.9]	[−2.5; 2.6]
RMSEC	0.55	0.38
RMSEP	1.34	1.31
